# Pragmatic trials in primary care. Methodological challenges and solutions demonstrated by the DIAMOND-study

**DOI:** 10.1186/1471-2288-7-16

**Published:** 2007-04-23

**Authors:** Gerdine AJ Fransen, Corine J van Marrewijk, Suhreta Mujakovic, Jean WM Muris, Robert JF Laheij, Mattijs E Numans, Niek J de Wit, Melvin Samsom, Jan BMJ Jansen, J André Knottnerus

**Affiliations:** 1 Research Institute Caphri, Department of General Practice, Maastricht University, PO Box 616, 6200 MD Maastricht, The Netherlands; 2 Department of Gastroenterology & Hepatology, Radboud University Medical Centre, Nijmegen, The Netherlands; 3 Julius Centre for Primary Care and Health Sciences, Utrecht University Medical Centre, Utrecht, The Netherlands; 4 Department of Gastroenterology & Hepatology, Utrecht University Medical Centre, Utrecht, The Netherlands

## Abstract

**Background:**

Pragmatic randomised controlled trials are often used in primary care to evaluate the effect of a treatment strategy. In these trials it is difficult to achieve both high internal validity and high generalisability. This article will discuss several methodological challenges in designing and conducting a pragmatic primary care based randomised controlled trial, based on our experiences in the DIAMOND-study and will discuss the rationale behind the choices we made. From the successes as well as the problems we experienced the quality of future pragmatic trials may benefit.

**Discussion:**

The first challenge concerned choosing the clinically most relevant interventions to compare and enable blinded comparison, since two interventions had very different appearances. By adding treatment steps to one treatment arm and adding placebo to both treatment arms both internal and external validity were optimized. Nevertheless, although blinding is essential for a high internal validity, it should be warily considered in a pragmatic trial because it decreases external validity. Choosing and recruiting a representative selection of participants was the second challenge. We succeeded in retrieving a representative relatively large patient sample by carefully choosing (few) inclusion and exclusion criteria, by random selection, by paying much attention to participant recruitment and taking the participant's reasons to participate into account. Good and regular contact with the GPs and patients was to our opinion essential. The third challenge was to choose the primary outcome, which needed to reflect effectiveness of the treatment in every day practice. We also designed our protocol to follow every day practice as much as possible, although standardized treatment is usually preferred in trials. The aim of this was our fourth challenge: to limit the number of protocol deviations and increase external validity.

**Summary:**

It is challenging to design and conduct a pragmatic trial. Thanks to thorough preparation, we were able to collect highly valid data. To our opinion, a critical deliberation of where on the pragmatic – explanatory spectrum you want your trial to be on forehand, in combination with consulting publications especially on patient recruitment procedures, has been helpful in conducting a successful trial.

## Background

Pragmatic trials are designed to investigate how effective a treatment strategy is in everyday practice [1]. The hypothesis and study design in pragmatic trials are developed specifically to answer questions of decision makers and should compare new with existing interventions in the indicated population using relevant health outcomes [2,3]. Researchers face a number of methodological challenges and need to make several choices in the design and conduct of pragmatic trials. This is especially true for primary care based trials where the broad spectrum of disease presentation and early clinical stage challenges the selection of an adequate study population. Though these challenges greatly influence the external and internal validity as well as the eventual significance of the study results, most publications do not elaborate on the choices made. This paper discusses several challenges in designing and conducting pragmatic primary care based trials we experienced in a large scale multicentre randomised trial on dyspepsia. This might be helpful for other researchers especially in the planning stage of new trials. Our objective is to contribute to quality improvement of pragmatic primary care based trials.

This paper will discuss three challenges in designing a study: choosing the right intervention and blinding treatment allocation, choosing an appropriate study population, and choosing the essential outcome measures. Subsequently the challenges in conducting a study will be discussed focusing on recruitment of participating general practitioners (GPs) and patients, and on dealing with protocol deviations. Each section will start with a brief introduction of pitfalls in general, followed by the rationale behind the choices made within the DIAMOND-study and a speculation of the consequences of our choices. The paper will end with conclusions describing the consequences of our choices for the expected usefulness and relevance of the DIAMOND results.

### The DIAMOND trial

**Figure 1:**
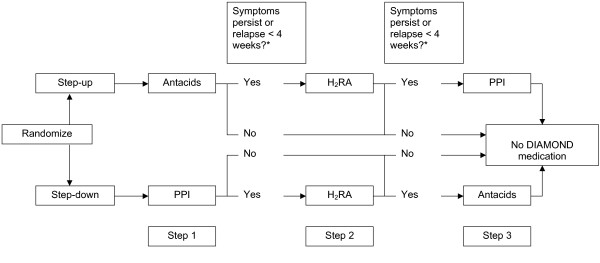
DIAMOND: Treatment strategies. * If the symptoms persisted the patient continued with the next treatment step. If the symptoms initially were relieved but relapsed within 4 weeks after stopping the treatment step, the patient also started the next treatment step. Otherwise (in case of a relapse after 4 weeks), the GP could treat the patient to their own judgement. Antacids (Algedrate-Magesiumoxide); H2RA: H2-receptor antagonist (Raniditine); PPI: Proton Pump Inhibitor (Pantozole).

*The Dutch study of InitiAl Management Of Newly diagnosed Dyspepsia (DIAMOND) investigates the effectiveness of two treatment strategies for dyspepsia: the step-up treatment strategy and the step-down treatment. The step-up treatment starts with antacids and, if the symptoms persist or recur, builds up to stronger medication, while the step-down treatment starts with the strongest drug (proton pump inhibitor (PPI)) and reduces stepwise to H2-Receptor Antagonists (H2RA) and antacids as long as the symptoms persist or recur. In *Table 1, 2, 3, 4 and Figure 1, 2* the design and research questions of the DIAMOND-study are described. The protocol of DIAMOND is registered on (identifier: NCT00247715) *[4]. *It is a pragmatic, large multicentre randomised controlled trial in primary care running from 2003 till 2007, in which 664 patients with dyspepsia were included and more than 300 GPs participated. The study is conducted with the joint expertise of three academic research centres from both primary and secondary care. While within DIAMOND besides effectiveness also cost-effectiveness will be analysed, this paper will focus on the evaluation of clinical end-points. Economic evaluation trials are facing specific methodological challenges, which are described for instance by Ramsey et al. and Tunis et al*. [3,5].

**Table 1:** The primary and secondary aims of DIAMOND

Primary aim of DIAMOND:
• To investigate which treatment strategy, "step-up" or "step-down" treatment, was the most (cost-)effective initial management strategy for patients with a new episode of dyspepsia in primary care.
Secondary aims of DIAMOND:
• To investigate which factors influence the severity of the GI complaints.
• To investigate which factors determine compliance with dyspepsia medication prescriptions and compliance with advised lifestyle changes.
• To investigate which factors influence treatment success.

**Table 2:** Inclusion and exclusion criteria of DIAMOND

1. Patients are included when they visit their GP for complaints of which the GP thinks that they originate from the upper GI tract and for which acid-suppressive medication can be effective.
2. Patients are included when they are 18 years or older.
3. Patients are excluded when they have used prescribed acid-suppressive medication in the last 3 months before inclusion.
4. Patients are excluded when they have had a gastroscopy in the year prior to inclusion.
5. Patients are excluded when they have alarming symptoms.
6. Patients are excluded when there are contraindications for prescribing acid-suppressive medication, such as pregnancy, liver or kidney malfunction.
7. Patients are excluded when they are not able to fill out (Dutch) questionnaires, for example because of language problems.

**Table 3:** DIAMOND inclusion and treatment protocol

**Figure 2:**
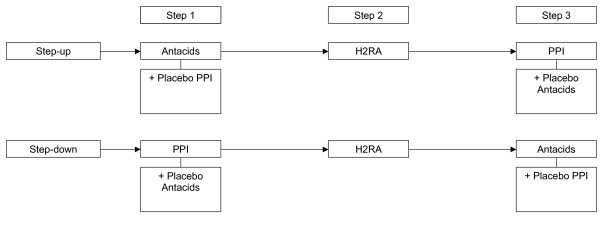
DIAMOND: Blinding of the treatment strategies. Antacids (Algedrate-Magesiumoxide); H2RA: H2-receptor antagonist (Raniditine); PPI: Proton Pump Inhibitor (Pantozole).

1. When a patient visits the GP, the inclusion and exclusion criteria are checked.
2. When the patient meets the criteria, the GP informs the patient about DIAMOND. When the patient wants to participate, he or she provides an informed consent.
3. The GP hands out the patient the medication for step 1. The medication is packed in boxes and is provided to the GP at the start of the study. Each box contains all the medication steps for one patient. The patient numbers on the boxes are linked to the numbers on the randomisation list in a sealed envelope kept at the researchers' office.
4. A blood sample is taken.
5. The patient receives the first questionnaire from the GP to fill out at home. Other questionnaires are sent to patients (Table 4).
6. The patient is treated according to the treatment protocol (see Figure 1 and 2). If the symptoms continue or relapse within 8 weeks after starting the medication step, the patient starts with the next treatment step. It is possible to shorten the treatment steps into less than 4 weeks, for instance when the patient suffers from side effects. The patient and GP are advised to schedule a follow-up visit at 4 weeks, which should be cancelled when the complaints are resolved.
7. When symptoms continue or relapse after medication step 3, the GP can treat the patient according to their own judgement.
8. The GP and the patient are informed six months after inclusion about the treatment allocation and the test results from the blood sample (whether the patient was infected with *Helicobacter pylori*).

**Table 4:** Measurements

Primary health outcome: Adequate symptom relief at 6 months according patients
Secondary health outcomes:
Severity of the GI complaints (at 2 weeks and after each treatment step)
Quality of life at 6 months (at 2 weeks and after each treatment step)
Additional research questions investigated:
- The cost-effectiveness of both treatment strategies.
- The association between genetic determinants and dyspepsia and treatment success.
- Compliance with prescribed medication advices and life-style advices and which factors influence compliance.
- The association between psychosocial determinants and dyspepsia and treatment success.
Self-administered questionnaires used:
- General questionnaire to measure effect of the treatment, costs, work absenteeism, demographical determinants, co-medication used and life-style.
- Gastrointestinal Symptoms Questionnaire; EuroQol 5D; SF 36; Compliance Questionnaire; SCL 90; Health Hardiness; Utrechts Coping List; Major Life Events

## Discussion

### Challenges in designing a study

#### Choosing the right intervention and blinding treatment allocation

Pragmatic trials evaluate the beneficial effect of a treatment strategy for clinical practice when applied by any clinician to any patient with the disorder studied. The intervention must be relevant and feasible to be generalised to clinical practice and it must be compared to the best available usual care (reference care). Randomisation and blinding caregivers, participants, and investigators for treatment allocation are used in trial settings to increase the internal validity and aims to ensure that an effect is solely caused by the intervention[6]. Inadequate blinding in trials proved to result in 30% lower odds ratios than adequate blinding[7]. However, in every day practice treatment is not blinded, and may be influenced by prejudices of GPs or patients. While blinding is important to increase internal validity, it may limit the generalisability of results. Furthermore, blinding treatment allocation is often difficult to achieve in pragmatic trials, because of differences in the appearance of treatment (for instance operation versus medication) or differences in the consultation scheme.

One possible solution is cluster randomisation[6], where one group of caregivers exclusively prescribes the experimental treatment and another exclusively the reference treatment. When all physicians within one centre are allocated to the same treatment arm, contamination will be reduced and all patients within one centre get the same treatment. Nevertheless, prejudices of caregivers, patients or researchers might still cause observation bias, for instance if the treatment is terminated preliminarily when physicians or patients do not expect the treatment to work. Although this reflects every day practice and might not be a problem in pragmatic trials (as long as patients are still included in analyses), observation bias decreases internal validity. Furthermore, because differences between caregivers can bias the results, one should then adjust for these differences with multi-level analysis.

#### The rationale behind our choices

*The DIAMOND project was designed to compare a step-up treatment strategy *(Figure 1) *(which is advocated in recent Dutch guidelines) with PPI-treatment (which is practised by many GPs). The appearances of both strategies differ too much to be suitable for blinding. Therefore, we decided to compare the step-up treatment strategy with a step-down treatment strategy, in which the PPI-treatment is followed by two treatment steps *(Figure 1). *Both treatment strategies were now made comparable in drug distribution and appearances by using placebos *(Figure 2). *This had several advantages; first, this design enables to investigate whether patients experience symptom relief on other (non-PPI) acid-suppressants when initial PPI-treatment fails. Second, PPIs can have a known rebound effect. In the step-down group it is possible to investigate whether patients, who initially responded well on PPIs but got a relapse, respond equally well on other (cheaper) acid-suppressants. Third, when patients needed all three medication steps, both groups received the same medication, only in a different order, so the influence of the order of medication on for example patient satisfaction can be investigated*.

*Our design also had some disadvantages. Our organisation of "step-down" treatment does not reflect usual care, which might affect generalisability. Some argued it is unethical to 'step-down' when the strongest drug is not effective. However, in our opinion patients can safely try the other two kinds of medication, before further investigation is established. Furthermore, in both groups patients had to use a placebo along with normal treatment. This can be a burden, since it means taking extra pills in step 1 and step 3, and it differs from everyday practice too*.

*Although heavily aimed for, we were not able to find completely identical placebos. However, patients would not be able to tell their treatment allocation and to ensure that GPs would not recognize the pills, non-transparent medication jars packed in sealed paper bags were used. Clustered randomisation as discussed above could have induced more bias as the treatment allocation would have been recognized easily by GPs after completing the first patient in their cluster*.

*We chose to disclose treatment allocation at 6 months, just after measuring primary outcome. We reached high internal validity at the cost of decreasing external validity. Primary outcome (adequate symptom relief according to the patient) was measured at 6 months, which could be 3–4 months after prolonged prescription of any medication chosen by the GPs after completing the trial. In usual care the GP would repeat prescription of the most effective on recurrence of the symptoms. However, because of the "late" disclosure of treatment allocation in DIAMOND, our GPs may have assumed that symptom relief may have occurred during the use of PPI and prescribed this after the trial medication was finished, while maybe the patient responded on the antacid. Consequently, blinding might have caused convergence of treatment after trial medication in both strategies, which decreases differences in measured effectiveness*.

*Infection with Helicobacter pylori can influence the effectiveness of treatment as well as relapse rates of symptoms. Therefore blood samples for serology were taken at baseline. The H pylori test results were also disclosed at 6 months to avoid the treatment or costs to be influenced by H pylori management before measuring primary outcome. Incidentally GPs requested to disclose H pylori test results earlier, in which case, the (theoretical) costs of H pylori testing were included for the cost evaluation of treatment. The medical ethics committee agreed with postponed disclosure since H pylori infection takes place early childhood and has no imminent association with the onset of symptoms. Early H pylori testing in this trial may have caused GPs to be more aware of H. pylori infection and may have urged them to inform about the test results more often than in normal practice. However, the alternatives, drawing blood samples only when a test is requested by the GP or after follow-up is completed, would have caused more drop-outs. The choice to communicate H. pylori test results at 6 months and take theoretical costs into account when requested sooner is a clear example of a way to control the treatment, while it probably decreases the external validity*.

*Our choices may all influence treatment effects. We believe that blinding the treatment allocation and the use of placebo led to more comparable treatment strategies, which probably led to a smaller difference between the true effects of both treatment strategies than in every day practice would exist*.

#### Choosing an appropriate study population

Regarding internal validity, according to Kleinbaum et al. selection bias is a distortion in the estimate of effect resulting from the manner in which subjects are selected from the target population [8]. Within DIAMOND all patients were randomly allocated to either the step-up or step-down treatment strategy, which makes selection bias unlikely.

Regarding external validity, it is very important that the investigated population should represent the target population, but how can optimal representation be achieved? First, the target population needs to be clearly defined by using inclusion and exclusion criteria. Second, the method of patient selection greatly influences representation (see "Patient recruitment"). The best way is to select patients randomly, but this is very challenging because it is difficult to avoid self-selection. Responding to an advertisement is a clear example of self-selection. Also GPs may be self-selected if they responded to an invitation letter to participate. This can be a problem when the participation of the GPs is associated with certain patient characteristics (education level, co-morbidity).

A representative patient sample must reflect all patients in the target population, including patients from minority groups, especially when treatment effects are supposed to be influenced by population characteristics. Translated questionnaires should enable immigrants to participate. Consideration should always be given to motivate patients expected to have low participation rates, for instance by tailoring patient information to gender or age.

There are several practical or judgemental reasons (lack of time, symptoms, preference, willingness) for a patient not to be included although eligible. Therefore, registration of all eligible patients and monitoring reasons for non-inclusion is preferred, to be able to judge inclusion selection. However, this is time consuming and researchers still would question the completeness of the registration. When available, electronic medical records might be helpful in estimating the proportion of non-included eligible patients. However, routine electronic medical records might also lack data to check eligibility (e.g. duration of symptoms) and won't always provide insights in the reasons for non-inclusion.

#### The rationale behind our choices

*We chose to focus on "adult patients with a new episode of dyspepsia", because the most effective treatment for these patients was unknown. Careful consideration with all the experts in the research board led to a limited number of inclusion and exclusion criteria to define these patients. The criteria were based on recent guidelines and were judged to be feasible and clear *(Table 2). *Regarding the representation of minority groups, it was not possible to make all relevant language adjustments, but translation from Dutch into English was provided. Some participating immigrants who spoke other languages had help from their relatives to fill out the questionnaires*.

**Figure 3:**
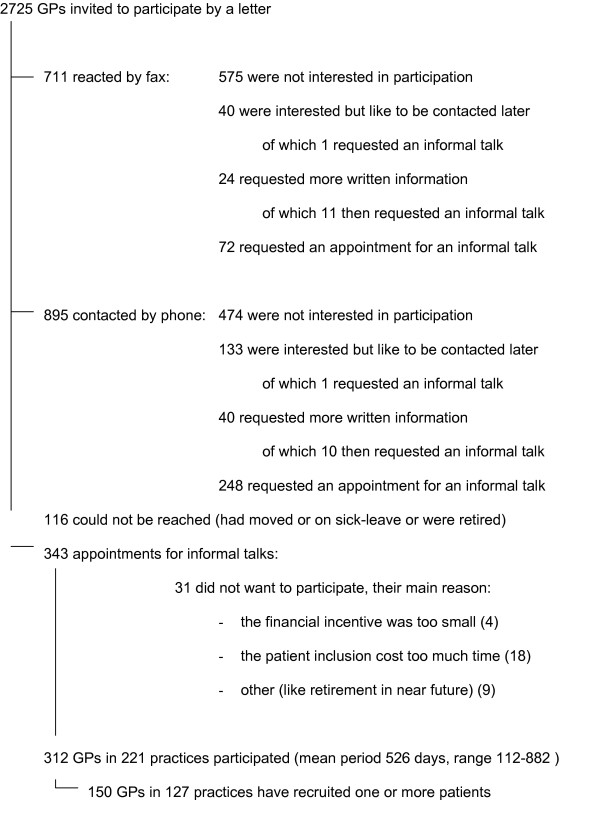
GP Recruitment.

*Patients were recruited by participating GPs. We invited as many GPs as possible within our geographic boundaries, resulting in 312 participating GPs distributed over the Netherlands *(Figure 3). *It is possible that especially GPs with a special interest in the gastrointestinal (GI) field were responding. This can be a problem if participation of the GPs is associated with effect modifying patient characteristics. However, it is likely that the heterogeneous group of participating GPs (GPs from urban as well as rural regions with solo, duo, or group practices) has resulted in a heterogeneous patient sample, which represents the primary care population*.

*To investigate initial treatment of patients with a "new" episode of dyspeptic symptoms, patients who used prescribed acid-suppressive drugs in the last 3 months were excluded. However, since patients with mild symptoms are more likely to be without medication for more than 3 months than patients with severe symptoms, this might have resulted in a patient sample with overrepresentation of patients with mildly severe dyspepsia. Moreover, maybe the GPs only invited patients with mildly severe dyspepsia, because they did not want to risk patients with more severe complaints to be treated with the step-up treatment strategy. Finally the representativeness of our sample will be investigated by comparing several relevant patient characteristics to results from other (preferably population based) studies*.

*Hypothetically, the difference in treatment effect between PPIs and antacids might be smaller in patients with mild symptoms. As a consequence the difference between the two treatment strategies might have been smaller than in every day practice where also patients with more severe complaints are treated*.

#### Choosing the essential outcome measurements

The value of study results is greatly determined by the definition of the primary outcome and choice of measurements. When the primary outcome is an objective measure, e.g. survival, it is easy to measure and define it. However, the outcome of many diseases in primary care needs more subjective evaluation, and selection and definition of the outcome may prove to be difficult. A proper definition can be based on literature or expert opinion. Furthermore, it needs to reflect what decision makers want to know. The endpoint also needs to be clear, and preferably comparable with other studies.

Concerning the measurements, the validity and reliability should always be critically assessed. To increase response rates questionnaires must be as short as possible. This is challenging, especially when several additional research questions are investigated as in our study (see Table 4). The additional value of every question in the questionnaire needs to be critically judged and a pilot study is preferred to estimate the feasibility and burden for GPs and patients.

#### The rationale behind our choices

*Choosing the primary outcome measure for DIAMOND was not easy because the presence or absence of "dyspepsia" can not be measured objectively*[9]. *Furthermore, dyspepsia is characterized by periods of remission followed by symptom relapse. We used "adequate symptom relief at 6 months, according to the patient" as primary outcome, following expert recommendations (Rome II criteria) and because this reflects the decision to stop or continue treatment in every day practice. It is generally accepted that symptomatic response can be used in dyspepsia because this is what GPs have to rely on in clinical practice. Besides, more objective measurements (e.g. endoscopy) poorly correlate with symptom severity. To enable a comparison with results from other studies we analysed the change in severity of the gastrointestinal symptoms and quality of life as secondary outcomes*.

*Additionally, choosing the right timing of the measurement of the primary outcome in a study with multi-step treatment strategies is difficult. Choosing a 6 month time interval is convenient for policy makers and feasible in trial practice. But the downside is that patients received trial medication for variable periods of time. Good responders may only have had the first treatment step, and if they remained symptom-free for 4 weeks after finishing treatment they did not start with the second treatment step. In case of relapse after 4 weeks or after finishing treatment step 3 treatment was left up to the GP. As mentioned above, primary outcome might be influenced more by the GP prescribed medication than study medication at the time of 6 months. This may have decreased differences between the treatment strategies at 6 months. We also measured short term outcomes (at 2 weeks, 4 weeks, etc.) to be able to determine the short-term efficacy of the individual treatment strategies*.

*We investigated the validity of the questionnaire for the severity of gastrointestinal complaints *[10,11]. *A pilot study among non-experts to investigate the burden of filling in our questionnaires showed that at baseline as well as at follow-up 15 to 30 minutes were needed for a complete response. This was judged to be acceptable and patients were informed of this time estimation before providing informed consent to participate*.

### Challenges in conducting a study

#### Patient recruitment

Many studies fail to recruit enough patients which compromise statistical power. A review by Mc Donald showed that only 31% of randomised controlled trials were able to reach their goals concerning patient recruitment [12]. There are several ways to recruit patients: from medical records, by advertisement or during consultation. The usage of medical records increases effective recruitment because it does not depend on patient presentation to recruiters during the inclusion period. However, this method can not be used when incident cases are required. Sellors et al. found barriers such as the availability of electronic medical records, the experience of office staff and GPs to produce patient sampling frames and ethical considerations [13]. Another method is patient recruitment via advertisements in (local) media or via flyers at the GP's office. However, patients responding to such advertisements may differ from patients not responding which leads to selection bias and hampers external validity. The conventional way to recruit patients is by the GP during consultation (incident cases). This way of recruitment approximates routine practice the most, which increases external validity. However, it poses a huge burden on the GP and is not always successful. There might simply be a lack of eligible patients or trial procedures can be too restrictive. According to Van Der Windt et al. the main reasons for not referring eligible patients to the research centre by participating GPs were: busy surgery hours, forgetfulness, or the conviction that a patient would benefit more from a specific intervention[14]. De Wit et al. found that successful patient recruitment in a dyspepsia trial was determined more by the motivation of GPs by the research group than by financial incentives, research topic, or research experience[15]. Foy et al. investigated in a meta-analysis the impact of interventions on patient recruitment and concluded that organisational characteristics (e. g. strong trial infrastructure) seemed to be important [16]. Furthermore, many interventions on patient recruitment were not evidence-based but based on the experience of the investigator [16].

Additionally, successful patient recruitment depends on the patients' motivation. Chang et al. found that the reasons for patients to participate could be divided into six general categories: 1) benefit to self; 2) benefit to others; 3) gratitude to the physician; 4) positive comments by the trusted professional; 5) the appearance, personality, manner and gender of the recruiter; 6) monetary compensation [17]. We agree with Chang that the most effective recruitment involves a direct and personal approach [17]. Patients appeared to enjoy being noticed and sorted out for something presented to them as important and special. The patient information and the GP need to address possible reasons and advantages for patients to participate.

#### The rationale behind our choices

**Figure 4:**
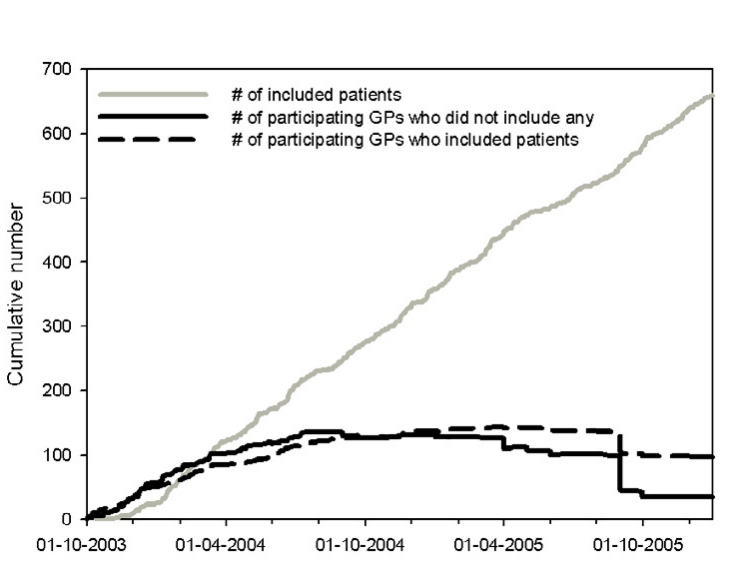
Patient recruitment and number of (successful) GP participants.

*Since we focused on patients with a new episode of complaints, we chose to recruit incident cases during consultations by the GP. To our experience successful patient recruitment depends on: 1) Close monitoring of recruitment statistics and extra measures to boost recruitment if necessary; 2) flexibility of the research protocol: it must be possible to adapt the protocol when GPs cannot use it in practice or when selection criteria are not clear or too strict; 3) good and regular contact with the GP or an assistant (preferably face-to-face or by telephone), which enables to remind and motivate them and notice and resolve difficulties. We visited the GPs after each new included patient to collect the patient's blood sample and provide new materials. The purpose of this visit was to reinforce the patient inclusion, but not to discuss how the included patient was treated to avoid an extra educational intervention. Furthermore, a monthly newsletter was sent to the GPs to remind them and to keep them posted. We tried to minimize the burden for the GPs and the assistants (for instance by taking blood samples ourselves when necessary) and answered questions promptly implying easy accessibility. Despite these efforts to motivate and assist the GPs, only 48% of the participating GPs recruited one or more patients *(Figure 4). *We can only speculate on the reasons for this disappointing number: maybe the inclusion and treatment was expected to be too time-consuming or maybe these GPs simply forgot to invite eligible patients despite of several reminders. Social desirability may have caused GPs to participate who were less motivated to include patients. Although ultimately successful, patient recruitment was very time consuming and needed sufficient budget for recruitment personnel. The intended inclusion period of two years had to be prolonged in October 2005 to include the desired number of patients. Only GPs who were expected to include several patients before the end of 2005 ("promising" GPs) were invited to continue patient recruitment. This explains the sudden fall in participating GPs in *Figure 4. *Interestingly, this did not decrease the patient inclusion in the last months, which suggests that it may be more efficient to only include highly motivated and "promising" GPs. Exclusion of reluctant GPs may hardly decrease inclusion rates but does decrease the workload for the researchers*.

#### GP recruitment

Patient recruitment in primary care based trials often depends on the cooperation of GPs. Since the demand on GPs to participate in research is growing and it is hard to keep the balance between research participation and daily practice, GPs must be very critical in their decision to participate[15]. Factors known to influence the physician's decision to participate include: 1) a personal interest in the research topic; 2) the relevance of the research question; 3) the personal connection with the researchers; 4) the collective ownership of the project; 5) the support of stakeholders or respected members of the professional community; 6) the revenue of costs associated with research participation; 7) the simplicity of protocols with low interference with patient care; 8) the availability of practice staff to assist the enrolment; 9) the timeliness of patient recruitment; 10) the satisfaction with study participation [18-20]. Van Der Windt et al. also mentioned that (accredited) postgraduate training is a reason for GPs to participate, and involvement in too many other studies is a reason not to participate[14].

A strategy for approaching primary care settings as proposed by Murphy et al. and Kocken et al. recommends identification of stakeholders and regional opinion leaders, using support letters by relevant professional organisations and supplying adequate, but concise, information [18,21]. It is important to consider and address the reasons for GPs to participate during the recruitment.

#### The rationale behind our choices

*For GP recruitment we wanted to invite as many GPs as possible within our geographical boundaries to gather a large heterogeneous GP sample. We retrieved the addresses of all eligible primary care settings from a registration at the three participating universities. The GPs received an invitation letter with information about the research together with a recommendation letter from the Dutch College of General Practitioners and the Dutch Institute for Healthcare Improvement. A reply form was offered to respond by fax. In case of non-response the GP was invited again by means of a telephone call. After an informal appointment at the GP's office, the GP decided whether or not to participate. For practical reasons the GP recruitment was spread out over the first period of patient inclusion. The results of GP recruitment are given in *Figure 4. *To our experience, however ultimately successful, the GP recruitment was very time consuming because of the many phone calls and visits. Although difficult, personal contact with the GP more positively influenced participation than leaving a message with the assistant. Spreading out the GP recruitment period gave us the opportunity to adjust the information letters and to approach more GPs to boost patient recruitment when the inclusion lagged behind. Our method of GP recruitment probably has resulted in a heterogeneous and representative relatively large GP sample, which is likely to have a positive influence on the generalisability of the results*.

#### Protocol deviations

Protocol deviation or protocol non-adherence by patients, GPs or researchers is common. Examples of protocol deviations are: drop-out, inclusion of ineligible patients, not receiving the allocated treatment, unplanned interruption or abortion of treatment; and not taking the trial medication as prescribed. Drop-outs are patients who stop their trial medication but remain available for follow-up [22]. Patients can also be "lost to follow-up", when they are no longer accessible to the investigators [22]. Eligibility errors are relatively common [22]. Objective eligibility criteria are less prone to error than subjective ones. If eligibility is checked before randomisation, the consequences of such errors will be minimal. However, in pragmatic trials commonly the eligibility is checked e.g. with blood measurements or patient self-reports, which are often only available after randomisation.

Bias can be introduced when protocol deviation affects both treatment groups differently [22]. Researchers therefore investigate whether the protocol deviation is caused by systematic or random errors, and whether it causes differences between both treatment groups. When protocol deviation is associated with one treatment arm (e.g. if the experimental treatment has more side-effects), it is important to take this into account because protocol deviations will also happen in every day practice. In a per-protocol analysis all patients with a protocol deviation will be excluded, which contrasts with the purpose of conducting a pragmatic trial [23]. Exclusion of patients can result in bias when the patients that stay included are no longer representative for the study population. Therefore, a per-protocol analysis is less suitable than an intention-to-treat analysis for pragmatic trials. Some pragmatic trials perform a per-protocol analysis additionally to an intention-to-treat analysis, but difficulties arise when both analysis produce different results. Whereas the results of a per-protocol analysis may provide additional insights in why a treatment has (or lacks) effect in every day practice, in pragmatic trials the intention-to-treat analysis is the way to determine the overall effect.

Protocol deviations can partly be prevented by writing simple and clear protocols, providing proper patient information, and by closely monitoring GPs and patients during a pilot study and adjusting the protocol if required.

#### The rationale behind our choices

*To reflect every day practice as much as possible we chose to write a flexible treatment protocol, in which for instance the GP was free to decide when patients could return for consultation (after 4 weeks was recommended) or how the consultation was done, by phone or personal. This has probably minimized our number of protocol deviations. We can only present some preliminary data at this moment, since not all analyses have yet been finished. No non-eligible patients were included. Eleven patients gave an informed consent but changed their mind shortly after and they did not start using our trial medication. One patient did not use medication step 1 for unknown reasons, but started medication step 2 approximately two weeks after baseline*. Table 5* shows the questionnaire response rates and suggests that number of patients "lost to follow" up was limited. For the intention-to-treat analysis, preliminary results indicate that for 98% of the patients the primary outcome at 6 months is present. We are able to achieve such a high response rate by contacting all non-responders or drop-outs by phone or via the GP (except for patients indicating not to be willing/able to participate anymore) and asking them to answer the question: has symptom relief been adequate since the start of the treatment? Most patients are willing to answer this single question*.

**Table 5:** Preliminary results*: the patient questionnaire response rates

N = 664*	Baseline	2 weeks	After step 1	After step 2#	After step 3#	6 months	1 year
Sent out	664*	613*	643*	595*	587*	659*	566*
Returned	629	543	525	474	454	646	373
Response rate	95%	86%	82%	80%	77%	98%	66%^

* Not all follow-up questionnaires were sent out, for instance when patients started step 2 within 2 weeks, or patients reported they no longer whish to receive questionnaires.

# if medication of this step was not started, questionnaires were sent out at 2 resp. 3 months.

^In case of non-response a reminder is sent out after all questionnaires except after 1-year, since this is an additional measurement to the original research protocol. This explains the low response rate.

*Some patients do not return the initial 6 month questionnaire, because they think that when their complaints are resolved they do not need to return questionnaires. To prevent this bias we send reminders pointing out the importance of always returning the questionnaire and contact non-responders by phone or via their GPs. The preliminary response rates for all questionnaires are given in *Table 5. *The response rates slowly decrease in time as can be expected. The length of the baseline questionnaire (T0) and the high number of questionnaires during the first month caused several patients to stop their participation. Although tested in a pilot study and explained in the patient information, this could not be completely prevented. Maybe in the near future easier ways to monitor complaints and retrieve important data (e.g. via the internet) will become accessible and can facilitate patient cooperation and prevent drop-out*.

### The consequences of our choices for the usefulness and relevance of the DIAMOND results

The results of this study are useful/relevant for policy makers, patients, GPs and researchers because a large population of well defined patients, which is generalisable to the Dutch population of patients with a new episode of dyspeptic symptoms. The study has a high internal validity because of the random treatment allocation, and the concealment of treatment allocation/blinding, which increases the value of the results for policy makers. However, the external validity is decreased by the use of step-down treatment instead of PPI-treatment (which is more common in every day practice) and by the blinding. Consequently, it is difficult to say what the effect of both treatment strategies will be if performed in every day practice.

In order to adapt the study protocol to routine daily practice, a multistep protocol was designed. Although this resembles everyday practice it makes analysis more difficult, because not all patients are in the same treatment step at a certain point in time, and because the period of time between finishing the trial medication and registration of the primary outcome may vary from patient to patient. In case this period is long, the primary outcome may be influenced by follow-up treatment chosen by the GP. This may decrease any differences between the treatment strategies, but on the other hand the primary outcome does provide essential information about the effectiveness of actual primary care treatment for dyspepsia. Furthermore, the differences between the two treatment strategies can be analyzed in more detail by analyzing the secondary endpoints (at 4 weeks, 12 weeks, etc...). Therefore, the trial design as presented will provide important insights in various strategies for treatment of dyspepsia in primary care.

## Summary

Pragmatic trials must ensure a high generalisability without compromising internal validity, which is very challenging [24]. Therefore, a critical appraisal of the planned design and method to conduct the trial before actually starting to collect data is essential. When several publications on patient recruitment or other pitfalls in designing/conducting a pragmatic trial are consulted, one may increase the likelihood of conducting a successful trial. Furthermore, it is very important to set priorities beforehand where on the 'spectrum from explanatory to pragmatic' you want your trial to be: do you want to know the "unbiased" effect of the treatment (as in explanatory trials) or are you more interested in the effects in daily primary care (as in pragmatic trials)? For instance, we chose to blind treatment allocation because otherwise prejudices of GPs, patients and researchers might have biased the results, although blinding contrasts with the purpose to reflect every day practice in pragmatic trials. On the other hand, we chose to use flexible treatment protocol to reflect every practice, what again might contrast with using standardized treatment in explanatory trials.

This paper shows that while we did not compare the two most frequently used treatment strategies in the DIAMOND-study, we were still able to collect highly valid data because of the blinded randomised treatment, the randomly selected heterogeneous patient sample and the research protocol that closely fits to normal practice. Although it is very difficult to recruit as many GPs and patients as needed, success can be determined by careful consideration of how the GPs and patients will be optimally recruited and what their reasons to participate or to refuse participation will be. Our experiences with the DIAMOND-study give an indication of what success rates regarding GP and patient recruitment and questionnaire response can be expected in similar studies.

## Competing interests

The author(s) declare that they have no competing interests.

## Authors' contributions

GF, CVM and SM designed the study, collected the data and performed statistical analysis. GF drafted the manuscript. JM, NDW, MN, RL, MS, JJ and JK participated in its design, conduct and coordination and helped to draft the manuscript. All authors read and approved the final manuscript.

## Pre-publication history

The pre-publication history for this paper can be accessed here:


